# Alpine Extremophytes in Evolutionary Turmoil: Complex Diversification Patterns and Demographic Responses of a Halophilic Grass in a Central Asian Biodiversity Hotspot

**DOI:** 10.1093/sysbio/syad073

**Published:** 2023-12-23

**Authors:** Anna Wróbel, Ewelina Klichowska, Arkadiusz Nowak, Marcin Nobis

**Affiliations:** Institute of Botany, Faculty of Biology, Jagiellonian University, Gronostajowa 3, 30-387 Kraków, Poland; Doctoral School of Exact and Natural Sciences, Jagiellonian University, Prof. St. Łojasiewicza 11, 30-348 Kraków, Poland; Institute of Botany, Faculty of Biology, Jagiellonian University, Gronostajowa 3, 30-387 Kraków, Poland; Botanical Garden, Center for Biological Diversity Conservation, Polish Academy of Sciences, Prawdziwka 2, 02-973 Warszawa, Poland; Botanical Garden of the Wrocław University, Sienkiewicza 23, 50-335 Wrocław, Poland; Institute of Botany, Faculty of Biology, Jagiellonian University, Gronostajowa 3, 30-387 Kraków, Poland

**Keywords:** Alpine biome, climate change, demography, distribution, diversification, Pamir Plateau, *Puccinellia pamirica*, single nucleotide polymorphisms

## Abstract

Diversification and demographic responses are key processes shaping species evolutionary history. Yet we still lack a full understanding of ecological mechanisms that shape genetic diversity at different spatial scales upon rapid environmental changes. In this study, we examined genetic differentiation in an extremophilic grass *Puccinellia pamirica* and factors affecting its population dynamics among the occupied hypersaline alpine wetlands on the arid Pamir Plateau in Central Asia. Using genomic data, we found evidence of fine-scale population structure and gene flow among the localities established across the high-elevation plateau as well as fingerprints of historical demographic expansion. We showed that an increase in the effective population size could coincide with the Last Glacial Period, which was followed by the species demographic decline during the Holocene. Geographic distance plays a vital role in shaping the spatial genetic structure of *P. pamirica* alongside with isolation-by-environment and habitat fragmentation. Our results highlight a complex history of divergence and gene flow in this species-poor alpine region during the Late Quaternary. We demonstrate that regional climate specificity and a shortage of nonclimate data largely impede predictions of future range changes of the alpine extremophile using ecological niche modeling. This study emphasizes the importance of fine-scale environmental heterogeneity for population dynamics and species distribution shifts.

Species diversification and demographic responses are deeply affected by climate changes and habitat distribution shifts (Hewitt 2000, 2004; Flantua et al. 2019). Particularly, profound environmental upheavals can strongly influence species spatio-temporal dynamics and genetic diversity (Dullinger et al. 2012; Lenoir and Svenning 2015; Steinbauer et al. 2018). Therefore, diverse and temperature-sensitive environments may especially favor complex eco-evolutionary processes, including major biological phenomena such as mass extinctions or the emergence of biodiversity hotspots (Qian and Ricklefs 2000; Antonelli et al. 2018; Mosbrugger et al. 2018; Flantua et al. 2019).

High rates of speciation and species range shifts are hallmarks of the alpine biome (Hughes and Atchison 2015; Muellner-Riehl 2019; Ding et al. 2020). Mountain regions have developed a rich and complex genetic legacy that could nowadays serve as an exciting platform to investigate mechanisms shaping biodiversity within a warming-sensitive arena. The extreme elevation gradients, rugged landscape relief, and diversified edaphic conditions have promoted profound eco-evolutionary changes in alpine ecosystems alongside glacial-interglacial cycles (Mosbrugger et al. 2018; Flantua et al. 2019; Rahbek et al. 2019a, 2019b). The environmental perturbations have played a major role in shaping population connectivity, providing recurrent opportunities for either isolation or gene flow, which are crucial elements along the speciation process (Abbott et al. 2013; Abbott 2019; Schley et al. 2022; Slovák et al. 2023). Intense climate changes repeatedly harassed mountain species, triggering strong and often contrasting responses among different geographic regions and ecological groups of mountain species (Elsen and Tingley 2015; Theodoridis et al. 2017; Yu et al. 2017; Liang et al. 2018; Carnicero et al. 2022; Guerrina et al. 2022). Although recent methodological advances have opened new avenues to reveal global determinants of species richness and diversification rates (Antonelli et al. 2018; Tietje et al. 2022; Cai et al. 2023), the impact of species-specific attributes and fine-scale environmental heterogeneity on population genomics still remains largely underexplored (Stein et al. 2014; Papadopoulou and Knowles 2016; Carnicero et al. 2022; Puglielli and Pärtel 2023). Evolutionary patterns are not yet fully uncovered at various spatial scales among organisms with different functional traits and life-history strategies.

Species dependent on limited habitat resources could be more fragile to environmental disturbances and prone to genetic erosion or even extinction (Aguilar et al. 2008; Haddad et al. 2015; Crooks et al. 2017). Therefore, alpine extremophiles may particularly experience abrupt ecological responses due to their heavy reliance on distinct and dynamically changing niches (James et al. 2005; Song et al. 2022; Sagwal et al. 2023). On the other hand, effective mechanisms related to stress response regulations and primary metabolic processes under limited resources (Eshel et al. 2021; Guo et al. 2021) could give the extremophiles the ability to survive adverse environmental changes projected for the future (Uzilday and Ganie 2023). Saline mountain wetlands are one of the most distinguished communities among extreme ecosystems and are landmarks of the arid and species-poor plateaus in the Andes and High Mountain Asia (Mianping 1997; Alonso and Rojas 2020). Nevertheless, the evolutionary histories of alpine halophiles as well as their future ecological potential remain poorly understood across the world’s mountain plateaus.

Here, we examined diversification and past demographic responses in an alpine salt-tolerant extremophyte using high-throughput DArTseq technology. As a model system, we chose *Puccinellia pamirica* (Roshev.) V.I.Krecz. ex Ovcz. & Czukav., a flagship Central Asian species of the high-elevation saline wetlands established on the Pamir Plateau (Ovchinnikov and Chukavina 1957; Sokolovskaya and Probatova 1976; Świerszcz et al. 2021). Strong habitat dependency, a restricted plateau-related distribution, and a diploid genome make *P. pamirica* an encouraging system in which to study species–environment interactions and their evolutionary consequences for the mountain extremophiles. We used the framework of molecular biogeography to reveal the fine-scale population structure of the model species and determine factors that may have shaped its past diversification. By integrating genomic results with paleoclimate data, we considered 2 demographic scenarios for the direction of population size changes in *P. pamirica* through the Late Quaternary. Specifically, we evaluated glacial expansion versus interglacial expansion hypotheses (Theodoridis et al. 2017) to uncover demographic trends of the cold-adapted halophile related to the high-elevation mountain plateau. Our aim was also to evaluate the importance of fine-scale landscape heterogeneity for the species ecological dynamics and limitations of niche projections under a prevalent shortage of nonclimate data. This study provides new insights into the processes shaping diversity and distributions of alpine species under the Late Quaternary climate changes.

## Materials and Methods

### Study System

**Figure 1.**
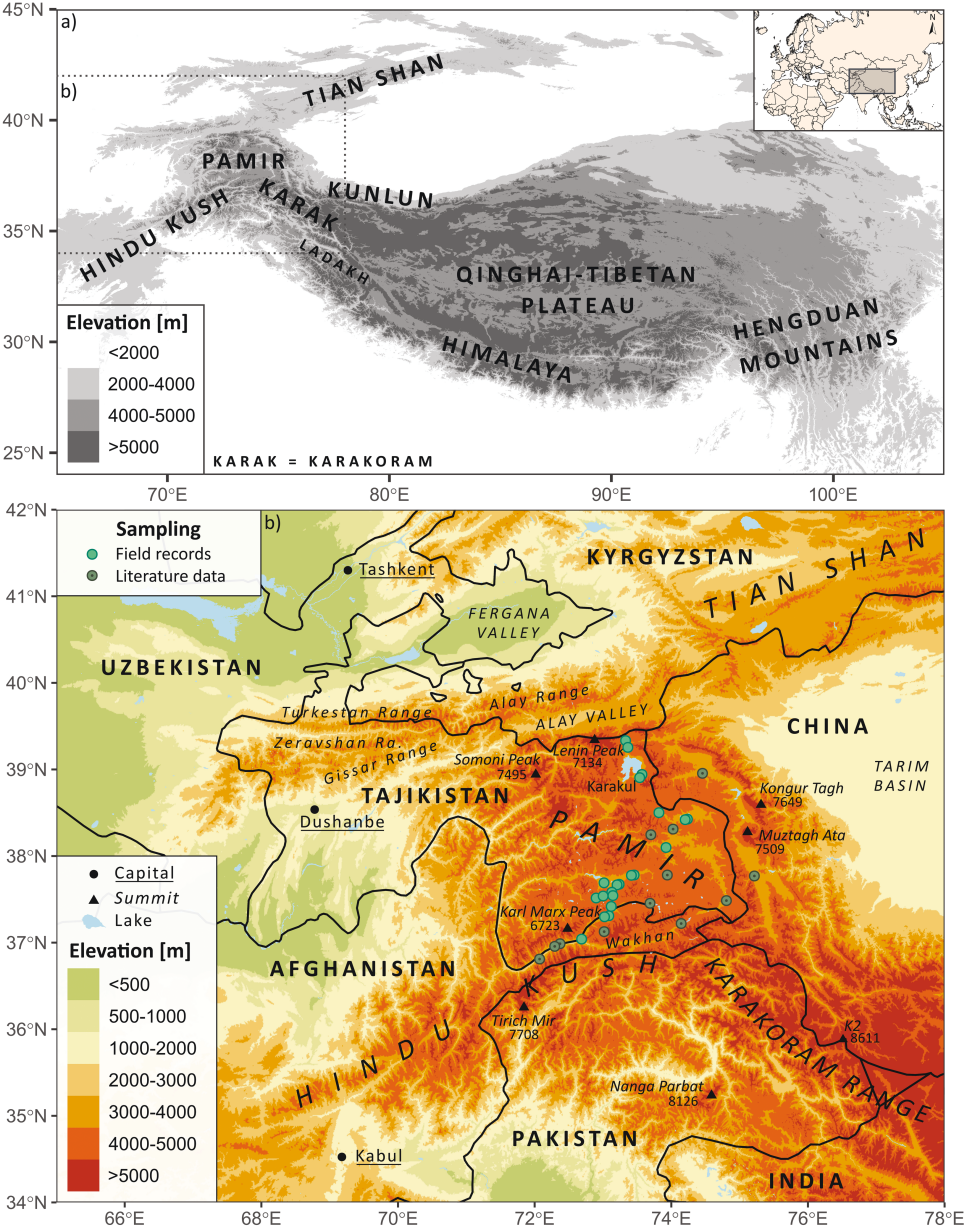
Study area: a) Location of the Pamir Mountains in High Mountain Asia; b) Sampling design for molecular and ecological analyses over the distribution range of a halophilic alpine grass *Puccinellia pamirica* on the Pamir Plateau and adjacent regions.

*Puccinellia pamirica* is a wind-pollinated diploid grass and a core component of the alpine halophilic vegetation (Supplementary Figs. S1.1 and S1.2, Dryad: https://doi.org/10.5061/dryad.sn02v6x85 [https://datadryad.org/stash/share/6Qvb_9E148Mtonvnf-2ygEI0g--NCwzxGMPiRtlUBtg]) as well as a flagship representative of saline alpine wetlands on the Pamir Plateau in the Eastern Pamir Mountains (Świerszcz et al. 2021), a part of the Mountains of Central Asia biodiversity hotspot (Mittermeier et al. 2004). The species tolerates hypersaline conditions and considerable water level fluctuations over the vegetation season. *Puccinellia pamirica* occurs in most of the major patches of saline wetland communities established on the Pamir Plateau, usually above 3500 m a.s.l. The species also grows below 2800 m a.s.l. in the lower Wakhan Corridor, situated near the southern edges of the platform (Fig. 1, Supplementary Tables S1.1 and S1.2). Throughout the article, we used the terms locality and population interchangeably.

### Sampling Design for Molecular Analyses

The primary aim of our sampling was to capture most of the species genetic background and landscape heterogeneity where halophilic vegetation occurs in the Pamir Mountains. We focused on maximizing geographic extent of our sampling rather than sample size per population because this approach could provide more power for revealing population genetic structure and factors that shape diversification (Prunier et al. 2013). Moreover, a considerable number of polymorphic markers, generated by the high-throughput SNP genotyping, could compensate for the small sample size per population (Landguth et al. 2012). In total, we collected 80 individuals for the genetic analyses, from 1 to 7 per 21 localities (Supplementary Table S1.1). Our sampling covers almost the whole known latitudinal range of the species in the Pamir Mountains and most of the major patches of the saline alpine wetlands established on the Pamir Plateau (Fig. 1).

### SNP Marker Discovery

Codominant single nucleotide polymorphism markers (SNPs) were generated by Diversity Arrays Technology (DArTseq) which combines complexity reduction methods, fragment size selection, and high-throughput sequencing, optimized for a target organism (Kilian et al. 2012). DArTseq resembles restriction-associated DNA sequencing (ddRADseq) (Peterson et al. 2012). However, it has the advantage of dealing with lower DNA quantities and lower quality DNA and assures lower allelic dropout rates (Sansaloni et al. 2011). The procedure for the SNP marker discovery and genotyping via the DArTseq platform for our model system is described in Supplementary Appendix S2. The SNP dataset was processed in the R environment (R Core Team 2021; RStudio Team 2021) using the dartR R-package with necessary dependencies (Gruber et al. 2018; Mijangos et al. 2022).

### Inference of Fine-Scale Genetic Patterns and Intraspecific Diversification

Principal Coordinates Analysis (PCoA) was performed to examine relationships between individuals of *P. pamirica* using the stats R-package (R Core Team 2021). Genetic structure was analyzed by means of the Bayesian clustering approach in Structure ver. 2.3.4 (Pritchard et al. 2000) using the admixture model with correlated allele frequencies. We used this technique to explore genetic variation in the model system and determine potentially distinct groups (clusters) of differentiated individuals as well as possible admixture fingerprints between these groups. The optimal *K* value was determined based on the estimated natural logarithm of the probability of the data (Ln Pr(X|*K*)) and the Δ*K* method (Evanno et al. 2005). Fixed and private alleles unique for an individual, a population, or a particular geographic group were calculated using the dartR R-package. The SNP filtering schemes used for the downstream analyses are described in Supplementary Appendix S3.

We used Treemix ver. 1.13 (Pickrell and Pritchard 2012) to reconstruct the species population splits and potential admixture events within the inferred network of population relationships. The program estimates the maximum likelihood tree of the examined populations, determines populations that are a poor fit to the model, and identifies migration events between these populations. We did not use an outgroup to root the ML tree in Treemix. We tested potential migrations edges (m) ranging from 0 to 20, using 10 replicates per each *m* value and 2 different block sizes (*k*) of 1 or 50 SNPs. Two different SNP filtering procedures used for the Treemix inference are described in Supplementary Appendix S4. Selection of optimal migration edges (number of potential gene flow events) was assessed using OptM ver. 0.1.6 (Fitak 2021).

### Demographic Reconstruction

Stairway Plot ver. 2.1.1 (Liu and Fu 2020) was used to infer the demographic history of *P. pamirica*. Past changes of the effective population size (*N*_e_) were reconstructed using the folded site frequency spectra (SFS; without knowing the ancestral alleles), calculated by means of the easySFS.py script [https://github.com/isaacovercast/easySFS]. We performed demographic analysis for the species (all individuals together) and the main genetic subgroups inferred in Structure. The folded SFS calculation procedure, Stairway Plot settings as well as model outputs based on different filtering strategies are included in Supplementary Appendix S5. The Stairway Plot was run assuming: (i) 2-years generation time which is the shortest period needed for the first filial generation in perennial grasses to reproduce, and (ii) a mutation rate of 6 × 10^−9^ per site per generation which is the estimated rate of spontaneous mutations in grasses—Poaceae (De La Torre et al. 2017).

### ABC for Demographic Inference

Approximate Bayesian computation (ABC) with supervised machine learning was performed using DIYABC Random Forest ver. 1.2.1 (Collin et al. 2021) to test 2 potential demographic scenarios for *P. pamirica*. We considered 2 major demographic hypotheses proposed for cold-adapted species (Theodoridis et al. 2017): (1) interglacial contraction, which assumes demographic expansion during the glacial period and decline during the interglacial, (2) interglacial expansion, which assumes demographic decline during the glacial period and expansion during the interglacial. As a warmer period, we assumed the Holocene since 11 ka (as 10–5.5 k generations), whereas for a colder period we defined the Last Glacial Period encompassing 11–115 ka (as 5.5 k–57.5 k generations) during the Pleistocene. The analysis was performed for all individuals pooled together. We used 20,000 simulations per scenario for model choice and 50,000 for parameter estimations. Both inferences were replicated 10 times using different reference tables (Chapuis et al. 2020). The scenario receiving the largest number of votes from the decision trees in the RF classifier was regarded as the most probable among the tested options. A detailed report of the DIYABC-RF analysis is included in Supplementary Appendix S5.

### Ecological Niche Model

The primary goal of this analysis was to reconstruct a network of potentially suitable habitats for *P. pamirica* in the Pamir Mountains, which could be used to evaluate the impact of habitat fragmentation on the genetic structure of our model species. We applied niche modeling during current conditions via MaxEnt ver. 3.4.4 (Phillips et al. 2006). We used all 33 known localities of *P. pamirica* across the Pamir Mountains with determined GPS coordinates (Fig. 1 and Supplementary Table S1.2). The model was calibrated on the species distribution range in the studied region extended by a buffer area of 200 km (VanDerWal et al. 2009). For the preliminary screening, we explored bioclimatic variables and the elevation layer from the WorldClim 2.1 database (Fick and Hijmans 2017), 6 soil-related categorical variables from the FAO Harmonized World Soil Database 1.2 (Fischer et al. 2008), bioclimatic and topographic variables from the Envirem database (Title and Bemmels 2018), and raster Euclidean distance to water calculated in ArcGIS 10.8 based on the selected water bodies from the customized OpenStreetMap waterway polygon layer of the studied area [https://www.openstreetmap.org; accessed via https://data.humdata.org/dataset/ with a keyword “Waterways” and country names].

We modeled the species niche in 30 arcseconds resolution (<1 km long square edge of the raster grid at the studied latitudes) to provide possibly the most accurate estimates of current habitat resources in the field. We also modeled the species niche in 2.5 arcminutes resolution (~3.7 km raster square grid) to acquire the approximation of a hypothetical scenario in which the species habitat connectivity is better than it is observed currently in the field—such lower raster grid resolution led to the overestimation of the potential distribution range of suitable habitats over the Pamir Plateau. See Supplementary Appendix S6 to learn more about the procedure for the downstream variable selection, MaxEnt settings, model advantages and limitations as well as past and future projections.

### Landscape Genetic Inference

Linear Mixed-Effect models (LME) were applied to evaluate which landscape components could explain the fine-scale population structure of *P. pamirica*. Our primary goal was to maximize the geographic extent of the research to provide the widest possible spatial coverage for our inference (Prunier et al. 2013). Therefore, we included all 21 sampled localities, regardless of their per-population sample size, and took an individual-based approach for the downstream analyses. LME models are considered a robust solution for individual-based landscape genetics. This approach enables us to evaluate several potential explanatory factors simultaneously and is a powerful tool to analyze complex datasets with repeated or clustered observations, which is a common data structure in the field of ecology and evolution (Shirk et al. 2018; Schielzeth et al. 2020). As potentially informative variables, we analyzed 3 selected isolation factors—geographic distance, habitat fragmentation, and isolation-by-environment. See Supplementary Appendix S7 for more details regarding the isolation factors.

We fit LME models using maximum likelihood in the lme4 R-package ver 1.1.26 (Bates et al. 2015) to test landscape genetic patterns. As a dependent variable, we considered the pairwise Nei genetic distance between sampled individuals (*D*_SNP_) calculated by the dartR R-package ver. 2 (Mijangos et al. 2022). As fixed factors, we tested geographic distance (*D*_GEO_—as a straight-line distance; *R*_GEO_—as a resistance factor), habitat fragmentation (*R*_HAB_—in 2.5ʹ and 30ʹʹ resolution), and isolation-by-environment (*D*_ENV_). To learn more about the selection of the pairwise distance metric, performance of the analyzed variables, model choice, and detailed output of the final best-fitted model, see Supplementary Appendix S7.

## Results

### Fine-Scale Genetic Patterns

**Figure 2.**
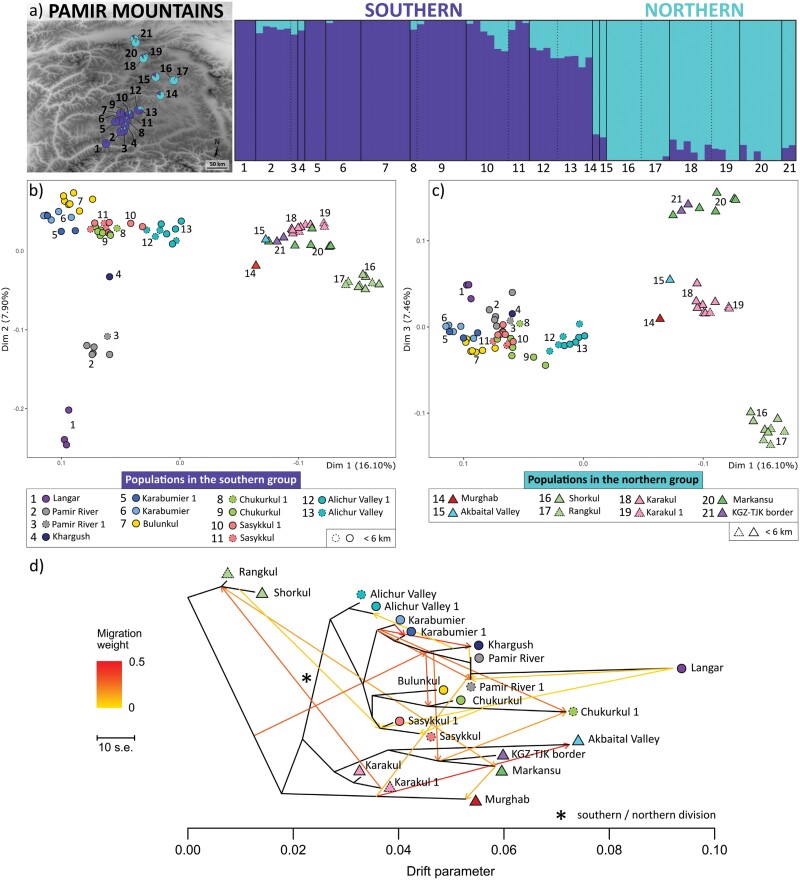
Spatial genetic patterns and diversification of a halophilic grass *Puccinellia pamirica* over the Pamir Mountains, Central Asia. a) Fine-scale population structure according to Structure for 2 clusters (*K* = 2)—pie charts on the map show mean *Q*-values within particular locality; bar plot represents *Q*-values in each individual (vertical line); locations in a distance up to 6 km are separated by dotted lines, more distant localities are separated by solid lines. b) Genetic similarity between individuals revealed by principal coordinates analysis (PCoA)—the second PCoA dimension primarily describes variation in the southern genetic cluster. c) Genetic similarity between individuals revealed by PCoA—the third PCoA dimension primarily describes variation in the northern genetic cluster. d) The Maximum Likelihood unrooted tree of population relationships with potential migration events inferred by Treemix; lines represent reconstructed gene flow and arrows indicate its direction.

**Figure 3.**
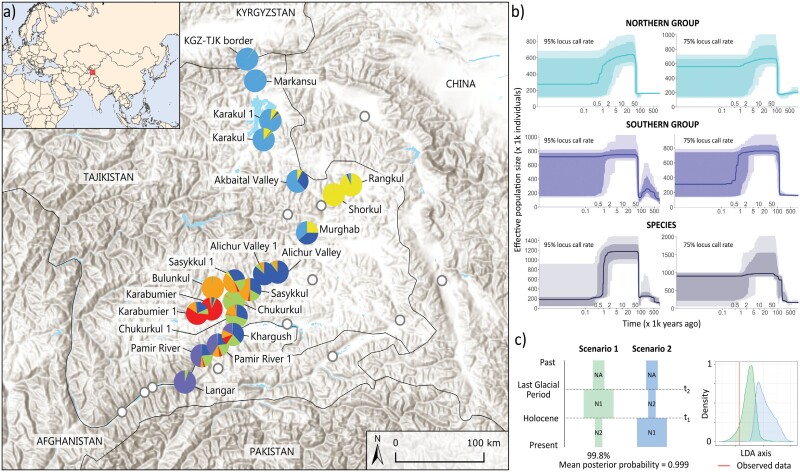
Spatial genetic structure and demography of the wetland-dependent halophilic grass *Puccinellia pamirica* in the Pamir Mountains, Central Asia. a) Fine-scale population structure according to Structure for 7 clusters (*K* = 7). b) Species demographic history inferred using the folded site frequency spectra in Stairway Plot; thick lines represent the median of the effective population size (*N*_e_) over time, surrounded by 75% and 95% confidence intervals; the time axes are on a logarithmic scale; models assume a spontaneous mutation rate of 6 × 10^−9^ estimated for grasses and a 2-year generation time. c) Species demographic scenarios tested in DIYABC Random Forest ver. 1.2.1; changes in effective population size (*N*) of the species are tracked over time (*t*) during particular climate periods in the past (*t*_1_, *t*_*2*_); parameter values are not to scale. Distribution map shows a topographic relief of the Pamir Mountains and the adjacent areas based on the Elevation/World Hillshade layer from Esri’s ArcGIS software [https://www.arcgis.com/home/item.html?id=1b243539f4514b6ba35e7d995890db1d].

We determined that *K* = 2 is the best *K* value to explain fine-scale population structure of *P. pamirica* according to the highest value of the Δ*K* statistic for Structure results (Supplementary Fig. S3.1). We found that the mean Ln Pr(*X*|*K*) reached a maximum value at *K* = 7, suggesting that *K* = 7 could also be considered as an optimal *K*. Structure revealed that the species demonstrates a major allele cline between its southern and northern geographic groups, which share a blurred boundary in the middle of the Pamir Plateau (Fig. 2a, Supplementary Figs. S3.2 and S3.3). The populations located near the center of the platform, in the Alichur Valley, demonstrated the lowest mean *Q*-values (<80%) according to Structure for *K* = 2. Similarly, the PCoA showed that the species populations are aggregated into 2 main genetic clusters, reflecting their geographic division in the middle of the Pamir Plateau (Figs. 2b and c). According to the PCoA, patterns of genetic similarity largely follow the spatial distribution of the species localities, whose genetic fingerprints are generally distinct from each other. The individuals of *P. pamirica* were primarily grouped with their relatives growing within the same wetland patch and were also closely aggregated with the neighboring localities situated in a distance of up to ~10 km. Likewise, Structure for *K* = 7 suggested large similarity among populations occupying shores of the same lake or river valley (Fig. 3a, Supplementary Fig. S3.4).

Treemix revealed a highly connected population network of *P. pamirica* throughout the Pamir Mountains, suggesting the potential occurrence of multiple migration events in the studied system (Fig. 2d, Supplementary Fig. S4.1). Because we did not use an outgroup, the network cannot be treated as rooted and its directionality should not be interpreted (e.g., the direction of introgression events). The inferred topologies generally reflect the spatial distribution of the species localities across the studied area. Three out of 4 Treemix models consistently supported the division into the southern and northern genetic groups using the same aggregates of populations as inferred in the PCoA and Structure (Fig. 2). However, Treemix remained more confused over the assignment of the populations located near the center of the Pamir Plateau, in the Alichur Valley. In one model, these populations were incorporated within the northern instead of the southern group (Supplementary Fig. S4.1).

We did not detect any private fixed alleles between the southern and northern groups of *P. pamirica* (Supplementary Table S3.1). Similarly, more than half of the examined populations did not have any private fixed alleles when comparing to all other populations. The discovered fixed alleles unique for a particular locality (~2% of the total dataset) occurred in most cases (~98%) in very small populations where we were able to sample only single individuals or in peripheral populations established along the southern or northern edges of the Pamir Plateau (Supplementary Table S3.1). Private alleles per individual accounted for 45% out of the analyzed polymorphic alleles, with a median value of 22 per individual (min = 1; max = 50), indicating a considerable share of the unique low-frequency variants across the whole species distribution range (Supplementary Table S3.2). However, 2 populations in the southern part of the Pamir Plateau (Bulunkul and Chukurkul) had largely decreased numbers of private alleles per individual (median value: 8 and 4, respectively) compared with other localities (median value: 25). According to Structure for *K* = 7, these 2 populations were also reconstructed as strongly homogenized in contrast to all other populations located in the southern part of the Pamir Plateau (Fig. 3a, Supplementary Fig. S3.4).

### Demographic Inference

The Stairway Plot models showed that *P. pamirica* experienced a large historical expansion followed by a recent decline over its demographic history (Fig. 3b). Similar trends were inferred for the species and its 2 main genetic subgroups. The SFS-based dating of the Pleistocene expansion differed depending on the SNP filtering strategy applied, ranging from ~20 k to ~100 k generations before the present (Supplementary Figs. S5.1 and S5.2). Time estimations for the demographic decline were much more consistent, showing that a decrease in the effective population size of *P. pamirica* occurred between several hundred years ago up to ~5 ka. The potential magnitude of the species decline during the Holocene varied among different filtering schemes and down projection strategies used for the Stairway Plot models, which determined either slight or large downtrends (Supplementary Figs. S5.1 and S5.2).

The ABC for demographic inference determined that glacial expansion of *P. pamirica* followed by interglacial decline (99.8 ± 0.4% of decision trees voting for the scenario) was a more probable model than glacial decline followed by interglacial expansion with posterior probability of 0.999 ± 0.003 (Fig. 3c, Supplementary Fig. S5.6, Supplementary Table S5.3). The Pleistocene expansion was dated back to 89,747 ± 3948 years ago (median value), ranging from 46,672 ± 4814 to 113,015 ± 633 according to the estimated 90% credibility interval of the parameter *t_2_* (Supplementary Fig. S5.8, Supplementary Table S5.4). The time estimations of interglacial demographic decline covered mostly the whole Holocene with the exclusion of the most recent 2000 years (Supplementary Fig. S5.9, Supplementary Table S5.5). The 90% credibility interval of the parameter *t*_1_ ranged from 2781 ± 325 to 10,666 ± 112 years ago.

### Ecological Niche Model

**Figure 4.**
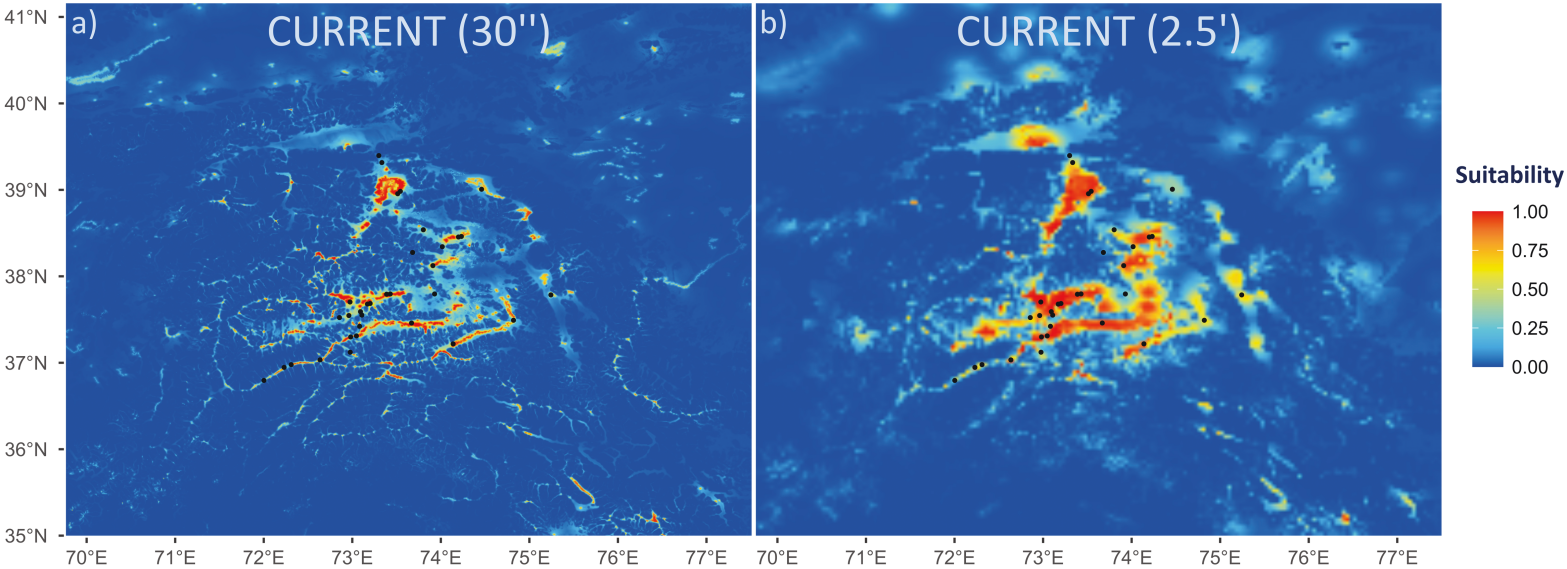
Ecological niche models of the wetland-dependent halophilic grass *Puccinellia pamirica* in the Pamir Mountains, Central Asia using MaxEnt ver. 3.4.4. Projection under current conditions based on six most informative variables—Euclidean distance to a water body (WaterDist), Envirem topographic wetness index (TopoWetness), Emberger’s pluviothermic quotient (embergerQ), mean monthly potential evapotranspiration of driest quarter (PETDriestQuarter), maximum temperature of the coldest month (maxTempColdestMonth), and mean monthly potential evapotranspiration of wettest quarter (PETWettestQuarter) modeled in: a) 30 arcseconds raster resolution and b) 2.5 arcminutes raster resolution.

Distance to a water body and topographic wetness index (a function of slope and catchment area reflecting potential topographic capacity of a terrain to store water) contributed the most to the inference of ecological niche for the wetland-dependent halophyte, irrespective of the additional climate variables used (Supplementary Fig. S6.1, Supplementary Tables S6.1 and S6.2). The most informative climate-related factors derived from the Envirem database that increased the model fit were: Emberger’s pluviothermic quotient (embergerQ), mean monthly potential evapotranspiration of driest quarter (PETDriestQuarter), maximum temperature of the coldest month (maxTempColdestMonth), and mean monthly potential evapotranspiration of the wettest quarter (PETWettestQuarter). The final 6-variable ecological niche model (ENM) under current conditions had a high average test AUC value indicating its good predictive power: 0.982 ± 0.026 for 30″ raster resolution and 0.973 ± 0.025 for 2.5ʹ resolution. The 30″ ENM suggested severe habitat fragmentation across the Pamir Plateau which well reflects the available wetland resources observed in the field nowadays (Fig. 4a), whereas the 2.5ʹ ENM recovered overall better population connectivity with only regional habitat fragmentation (Fig. 4b).

All 33 analyzed locations of the major wetlands in the Pamir Mountains, where *P. pamirica* occurs, could currently be characterized by extremely arid conditions with a median rainfall of 58 mm during the wettest quarter (min = 28 mm, max = 115 mm; bio16) and 32 mm during the warmest quarter (min = 14 mm, max = 70 mm; bio18), which represents very low precipitation rate considering the Pamir Mountains and their adjacent areas (Supplementary Fig. S6.5, Supplementary Table S6.8). Populations of the model species reach moderately cool temperatures during the warmest quarter with a median value of 11 °C (min = 7 °C, max = 19 °C; bio10). Most optimal habitats for the wetland-dependent halophyte extend nowadays near scattered lakes or river banks in the Alichur, Pamir, and Aksu river valleys in the southern part of the plateau as well as along the shores of Rangkul-Shorkul and Karakul terminal lakes in the northern part of the plateau. There are only a few potentially suitable regions for *P. pamirica* located outside the plateau. Due to terrain roughness, limited saline wetland niches are restricted to narrow floodplains along the rivers descending from the plateau. Langar (Population 1) was the only population of *P. pamirica* that we were able to rediscover along the Panj River valley in the lower Wakhan Corridor, remaining outside the Pamir Plateau below 3000 m a.s.l. Multiple isolated patches of potentially favorable habitats for *P. pamirica* demonstrate regional island-type patterns and their huge fragmentation is currently visible across the whole species distribution range in the Pamir Mountains (Fig. 4a).

### Landscape Genetic Inference

The LME models supported a multifaceted relationship between isolation factors and the spatial genetic structure of the Pamir halophyte. The best model included geographic distance, isolation-by-environment, and habitat fragmentation (Supplementary Table S7.8). The first-ranked model explained more than 50% variance through its 3 fixed factors and 77% variance overall. All 3 explanatory factors did not show any substantial multicollinearity (VIF < 3.7), were significantly associated with genetic distance at *P* < 0.001, and had similar predictive contributions with a slight predominance of R_HAB-2.5ʹ_ as shown by beta weights (Supplementary Table S7.9).

All 33 populations of *P. pamirica* are spread over a distance of 3–327 km. Among 21 locations sampled for genetic analyses the highest pairwise geographic distance reaches 269 km (Supplementary Table S7.1). Such spatial isolation explained a large amount of genetic variation in *P. pamirica* (Supplementary Table S7.9).

The PCoA, PCA, and Niche Equivalency Test uncovered patterns of environmental variation among the species localities established in the Pamir Mountains (Supplementary Fig. S7.1). The southernmost populations along the Panj and Pamir River floodplains (Populations 1–3) were distinguished by the highest temperatures and lowest precipitation during the warmest quarter. The next group northwards covered much higher elevations (Populations 4–6, 8, 9), between 3900 and 4200 m a.s.l., and included the uppermost sampled locality at 4193 m a.s.l. below the Khargush pass (Population 4). The next aggregation, in the central part of the mountain system encompassing wetlands in the Alichur River catchment area (Populations 7, 10–13), represented a moderate environmental character among all populations. The adjacent group, consisting of Murghab locality, Rangkul, and Shorkul lakes (Populations 14, 16, 17), was primarily distinguished by the lowest precipitation and highest temperatures during the wettest quarter. The northernmost cluster including populations in the Akbaital River valley, Karakul basin, and Trans-Alay Range (Populations 15, 18–21), were located between 3900 and 4150 m a.s.l. and differentiated by the highest precipitation during the warmest quarter (Supplementary Tables S7.2 and S7.3). Isolation-by-environment was recovered as an important contributor to explaining the observed patterns of genetic variation in *P. pamirica* (Supplementary Table S7.9).

Higher interpopulation connectivity derived from the 2.5ʹ ENM was better fitted to the patterns of genetic variation than larger habitat fragmentation modeled in 30″ resolution (compare Figs. 4a and b as well as performance between *R*_HAB-2.5ʹ_ and *R*_HAB-30″_ in Supplementary Table S7.8). The LME models determined that the species genetic structure better relates to higher interpopulation connectivity than currently offered by the wetland resources available in the Pamir Mountains.

## Discussion

### Fine-Scale Population Structure

We detected a complex evolutionary history of the alpine halophyte across the Pamir Mountains. The ΔK statistic computed for the Structure results supported the occurrence of 2 subgroups of *P. pamirica* divided by a clear allele cline in the center of the Pamir Plateau. Structure identifies the genetic clusters based on the detected variation in allele frequencies and does not require a predefined population assignment (Pritchard et al. 2000). However, the ΔK method cannot identify *K* = 1 as the best option to resolve the correct number of genetic clusters (Evanno et al. 2005), leading to a potential bias towards excessive selection of *K* = 2 (Janes et al. 2017; Cullingham et al. 2020). Nevertheless, we determined that the general pattern of the northern/southern division was also reconstructed in the PCoA and Treemix models. Despite the lack of fixed differences detected between these 2 intraspecific groups, the center of the Pamir Plateau appears to define the top level of fine-scale population structure in the studied alpine halophile.

Hierarchical population structure and uneven sampling across the putative subgroups could interfere with the optimal representation of the species genetic subdivision (Puechmaille 2016). Particularly, in the case of isolation-by-distance, in which allele frequencies tend to change gradually across the studied area, the selected value of *K* and further group assignment may be arbitrary in Structure (Pritchard and Wen 2002). As a consequence, the analysis may incorrectly support a larger number of distinct groups than are actually present, as observed in the case of *K* = 7 for *P. pamirica*. We showed that the spatial distribution has played an important role in shaping population relationships of *P. pamirica*, promoting mostly gradual changes of evolutionary relationships among the studied localities. Although we found evidence for detectable genetic differentiation among the examined populations, it was not sufficient to delimit distinct regional genotypes of the alpine halophile across the Pamir Mountains. Accordingly, our Structure results for *K* = 7 suggest shared ancestry between many populations of *P. pamirica*, which could be the consequence of shared history or admixture rather than strong intraspecific diversification.

Complex reticulate histories might be difficult to reconstruct when they involve multiple gene flow events entangled along various time horizons (Fitak 2021; Slovák et al. 2023). Moreover, the directionality of evolutionary changes cannot be interpreted within the unrooted population network of *P. pamirica*. The reconstruction of the network topology and the number of migration edges varied among Treemix models based on different SNP filtering strategies and model block sizes. This inconsistency shows that inferring the placement of admixture events in the population network of *P. pamirica* is difficult given the high levels of connectivity between the species localities. Therefore, the topologies and migration events inferred in Treemix provide an approximation of these complicated population relationships.

Our model system shows how an alpine species could develop complex fine-scale population structure even within a single mountain range. Recurrent divergence and mixing phases rather than long-term isolation are one of the key drivers boosting genetic diversity and emergence of rich mountain biota (Mosbrugger et al. 2018; Flantua et al. 2019; Muellner-Riehl 2019). The alpine biome is particularly renowned for high diversification rates (Hughes and Atchison 2015), which may still remain independent from species richness (Tietje et al. 2022). Our case study reveals that a species-poor alpine region such as the Pamir Plateau could still develop a rich evolutionary history at the intraspecific level.

### Landscape Genetics

We showed that fine-scale environmental heterogeneity rather than climate primarily determines the distribution patterns of the alpine halophile. Therefore, the lack of complete high-resolution abiotic data throughout Central Asia, particularly for soil properties (Ivushkin et al. 2019; Batjes et al. 2020), largely limits the capacity to infer ecological background for the development of population genetic structure in *P. pamirica*. Moreover, multicollinearity between potentially explanatory variables remains another inherent constraint in spatial genetics (Prunier et al. 2015). Specific causality between single environmental predictors and genetic variation is difficult to disentangle given the shared effects on a response variable among collinear factors (Graham 2003; Dormann et al. 2013). Therefore, we analyzed only 3 selected and ecologically different mechanisms within a distance-based framework as a trade-off to investigate species-environment interactions under detected methodological limitations.

The LME models showed that isolation-by-distance has strongly influenced fine-scale population structure of *P. pamirica*, similarly as in the case of many other alpine plants occupying large areas in High-Mountain Asia (Yu et al. 2019). Moreover, detectable regional features of the Pamir landscape could potentially stimulate local genetic differentiation, because isolation-by-environment explains a part of the genetic variation observed in *P. pamirica*. These findings add to the growing evidence that potential effects of adaptations to microniches could also be the essential drivers of genetic differentiation among the alpine species in addition to isolation-by-distance (Wu et al. 2019; Van Buskirk and Jansen van Rensburg 2020; Zhao et al. 2020). Moreover, spatial patterns of genetic variation in *P. pamirica* are better fitted to higher landscape connectivity than currently offered by the largely fragmented wetland network across the deserted Pamir Plateau. Such discrepancy could be attributed to rapid aridification and regional decline of hydrological network in the plateau ecosystem during the Late Holocene (Sagwal et al. 2023), which has not yet become fully incorporated in the genetic structure of the alpine halophyte. Alternatively, efficient wind-driven dispersal of diaspores (Soons et al. 2004) may provide enough capacity to regionally compensate for habitat fragmentation in the studied graminoid species considering high-velocity winds in the Pamir Mountains (Mętrak et al. 2015). As a result, many populations of *P. pamirica* could still remain more connected than the contemporary landscape suggests.

### Demographic Responses

Correct inferences on past demographic history are a huge challenge in natural populations, which usually depart from the idealized Wright–Fisher model. Specifically, unaccounted population substructure may lead to spurious reconstructions of past changes in the effective population size (Ptak and Przeworski 2002; Städler et al. 2009; Orozco-Terwengel 2016). Violations of the equilibrium conditions could particularly generate incorrect signals of population decline (Chikhi et al. 2010; Heller et al. 2013). Moreover, the interpretation concerning an overrepresentation of rare alleles could also be confounding. Although an excess of low-frequency variants is often a fingerprint of demographic expansion (Keinan and Clark 2012), other processes could also be responsible for the development of such genetic patterns (Nielsen 2005; Eldon et al. 2015; Marchi and Excoffier 2020; Freund et al. 2023). Apart from the abovementioned caveats, other essential factors such as genomic data type and filtering strategy, desired timeline of past inference as well as number of sampled individuals also affect the performance of demographic reconstructions in nonmodel organisms (Huang and Knowles 2016; Beichman et al. 2018).

We showed that 2 main species subgroups, northern and southern, demonstrated similar SFS-based demographic trends as the species in general. Thus, for simplicity, we performed the ABC analysis for the entire species, without accounting for fine-scale population structure and potential admixture events between the species genetic subgroups. The ABC approach could be the most efficient while analyzing a limited number of contrasting models focused on major demographic events (Cabrera and Palsbøll 2017). Therefore, we tested 2 main hypotheses proposed for the responses of cold-adapted plants during the Late Quaternary (Theodoridis et al. 2017), which allowed us to reduce the number of candidate scenarios. The skyline plots and ABC for demographic inference suggested that glacial expansion followed by interglacial decline could characterize the evolutionary history of *P. pamirica* while considering solely the potential changes in its effective population size.

Cold-adapted species, including alpine and arctic organisms, experienced huge demographic turbulences and distribution shifts during the Quaternary glacial-interglacial cycles (Hewitt 2000, 2004). In light of current global changes, the topic remains particularly relevant to better understand processes shaping diversity within potentially the most fragile and warming-sensitive ecosystems (Lenoir et al. 2010; Dullinger et al. 2012; Steinbauer et al. 2018). Notably, climate-related species range shifts and their evolutionary consequences still remain largely underexplored across key mountain regions that harbor a large proportion of the world’s alpine biodiversity (Lenoir and Svenning 2015). Two major hypotheses were proposed to explain the responses of cold-adapted species to climate oscillations (Theodoridis et al. 2017). The interglacial contraction hypothesis (or glacial expansion hypothesis) postulates that cold-adapted species demonstrated larger distribution ranges during colder periods by spreading across ice-free peripheral or lowland regions. During warmer periods these species tend to retreat to interglacial refugia towards higher latitudes or mountain summits (Stewart et al. 2010; Flantua et al. 2019; Vintsek et al. 2022; Dantas‐Queiroz et al. 2023; Nobis et al. 2023). This hypothesis suggests that warming could generally threaten the alpine species by aggravating habitat loss and shifting climate conditions beyond the ecological capacity of the cold-adapted organism (Parmesan 2006). On the other hand, the interglacial expansion hypothesis (with a special case of postglacial expansion) assumes that some cold-adapted species were not able to spread far from the glaciated areas during colder periods due to low competitiveness, dispersal barriers or shortage of suitable habitats (Theodoridis et al. 2017). Only upon temperature increase and glacier retreat, the species were able to expand their distribution ranges into the higher elevated areas (Birks 2008; Stewart et al. 2010). The expansion after the Last Glacial Period has been revealed in selected narrow mountain endemics (Tribsch 2004; Carnicero et al. 2022), facultative alpine species (Theodoridis et al. 2017), and species occurring on the north-eastern Qinghai-Tibetan Plateau (Zhang et al. 2005; Meng et al. 2007; Yu et al. 2017). The interglacial expansion scenario has recently received much more attention due to the potentially favorable impact of temperature rise on the selected species associated with the mountain plateau ecosystem (Elsen and Tingley 2015; Liang et al. 2018).

We determined that the explosive increase in the effective population size of *P. pamirica* could coincide with the Last Glacial Period. The historical expansion of the alpine halophile may align with the most extensive glacier advances in the Pamir Mountains estimated between 50 and 100 ka (Owen and Dortch 2014). The local Last Glacial Maximum preceded the global LGM at ~21 ka as increasing aridity was most likely responsible for the limited ice and snow accumulation in the Pamir Mountains during later Pleistocene glacial periods, including the global LGM (Abramowski et al. 2006; Owen and Dortch 2014). Nevertheless, arid and cold plateau regions in Central Asia were most likely not suitable for plant species during glacial periods, as proposed for the north-eastern Qinghai-Tibetan Plateau (Yu et al. 2017). In that region, plants could (re)colonize the high-elevation platform only upon postglacial temperature rise. Such distribution changes could also characterize the cold and arid ecosystem of the Pamir Plateau over the Late Quaternary. Nevertheless, the putative spatial range shifts associated with past demographic changes of *P. pamirica* are difficult to decipher (See: *Past Projection* in Supplementary Appendix S6).

The potential postglacial (re)colonization of the Pamir Plateau did not coincide with demographic expansion of *P. pamirica* after global LGM according to our demographic Stairway plot and ABC inferences. Moreover, recurrent climate oscillations over the Pleistocene did not leave fingerprints on the species SFS or at least they remained beyond the inferential power of the Stairway Plot method (Liu and Fu 2015). Presumably, the alpine halophile could have demonstrated its maximum distribution range during local LGM in accordance with the glacial expansion hypothesis (Theodoridis et al. 2017), spreading across potentially suitable habitats in lower-elevated flat basins which are in close proximity to the Pamir Mountains (see: *Past Projection* in Supplementary Appendix S6). The currently observed distribution extent would therefore be a reduction compared with the former wider range occupied during cooler interval. This could also explain the demographic decline reconstructed over the Holocene, linking it with the potential upslope dispersal and interglacial range contraction. Nevertheless, the recent decrease in the effective population size could also be attributed to the effect of isolation-by-distance and/or habitat contraction (Leblois et al. 2006), which both affect the alpine halophile according to our LME models. Particularly, small and declining populations could react strongly within a fragmented landscape and are much more prone to loss of genetic diversity or even extinction (Melbourne and Hastings 2008; Haddad et al. 2015). Moreover, the populations remaining at the distribution margins are potentially more fragile to climate-related transformations and human pressure (Wiens 2016). Such individualistic population responses (Bennett and Provan 2008; Angert et al. 2011; Wessely et al. 2022) could also play a major role in shaping the genetic pool and demography of *P. pamirica* in the future.

### Eco-Evolutionary Dynamics of the Alpine Halophile—Future Implications

Strong distribution shifts and progressing habitat fragmentation could trigger regional declines, leading to the species genetic impoverishment and local extinctions (Meza-Joya et al. 2023). The majority of alpine plants are expected to experience profound range changes and population contractions in response to climate warming (Lenoir et al. 2010; Chen et al. 2011; Dullinger et al. 2012; Steinbauer et al. 2018). In the mountains with a typical pyramid shape, the available area for occupancy declines at higher elevations so that the alpine species escaping upslope may eventually face complete habitat depletion (McCormack et al. 2009; He and Jiang 2014; Chen et al. 2019). On the other hand, high-elevation flat plateaus could potentially provide refugial space for the mountain species upon temperature rise (Elsen and Tingley 2015; Liang et al. 2018). The observed regional greening effect and vegetation transformations across the Asian plateaus suggest ongoing distribution shifts of plant species in this largely diverse and often harsh ecosystem (Teng et al. 2021; Lou et al. 2023). Particularly, extreme climate conditions, complex geomorphic processes, and intrinsic species ecological traits are among numerous factors which could strongly affect species dispersion towards and across mountain regions (Wang et al. 2006; Macias-Fauria and Johnson 2013; Alexander et al. 2018; Rull and Vegas-Vilarrúbia 2020; Na et al. 2021; Carnicero et al. 2022; Dantas-Queiroz et al. 2023). Moreover, the unprecedented speed of current climate changes could pose a serious threat to highly specialized alpine organisms, hindering potential adaptive readjustments and limiting range shift opportunities (Wiens and Graham 2005; Dullinger et al. 2012; Corlett and Westcott 2013; Moritz and Agudo 2013).

Desertification has been commonly observed in drylands upon global warming (Huang et al. 2016; Burrell et al. 2020). Nevertheless, arid alpine ranges such as the Pamir Mountains exhibit much more complex responses to temperature rise compared with water-stressed lowlands (Luo et al. 2018; Wang et al. 2018; Liu et al. 2019; Li et al. 2022). Apart from the levels of net precipitation, terrestrial water storage in desert alpine basins is also heavily reliant on influxes from neighboring areas and underground supplies. Therefore, warming-related glacier melting and permafrost thawing could stabilize or even improve water availability at high elevations, regardless of low precipitation levels and/or increasing evaporation (Liu et al. 2019; Mętrak et al. 2019, 2023a; Song et al. 2022). Although this mechanism may compensate for the desertification threat in the short run, these water supplies might be depleted in the longer perspective (Mętrak et al. 2023b). Lake and wetland responses vary among different regions, depending largely on the complex interplay between disturbances in local climate, hydrosphere, cryosphere as well as human activities (Song et al. 2014; Satgé et al. 2017; Zhang et al. 2017, 2020; Yoshikawa et al. 2020; Sagwal et al. 2023). Consequently, the cumulative fine-scale environmental changes rather than the climate itself (Suggitt et al. 2011; Sandzewicz et al. 2023) could be a primary factor shaping population dynamics and may determine the ultimate fate of the wetland-related alpine halophile.

Precipitation patterns, glacier mass budgets, and runoff trends remain region-specific and cannot be easily generalized throughout Central Asia (Immerzeel et al. 2010; Luo et al. 2018; Na et al. 2021). Due to lack of consensus on future climate changes as well as complex hydrological trends and glacier dynamics revealed over the Pamir Mountains (Gardelle et al. 2013; Kääb et al. 2015; Knoche et al. 2017; Heinecke et al. 2018; Mętrak et al. 2023a), it is difficult to predict further population dynamics of the halophilic species based on niche modeling (see: *Advantages and Limitations of Niche Models* and *Future Projection* in Supplementary Appendix S6). We presume that slight improvement in water availability throughout the Pamir Plateau could favor *P. pamirica* to expand its distribution range through newly established water reservoirs. On the contrary, largely increasing humidity could intensify competition pressure from nonextremophilic species, whereas strong desertification might trigger local extinctions and further disrupt the habitat network of *P. pamirica*. Upon further temperature rise, drought and salinity tolerance may give a temporal advantage to halophiles compared with other wetland-related species (Mętrak et al. 2023a) unless the water resources disappear completely. In the least optimistic scenario, limited patches of plateau-related wetlands could become the equivalent of the mountain top “sky-islands” and turn into the “nowhere to escape” traps (Chen et al. 2019; Muellner-Riehl 2019). Halophilic wetland species established within extremely arid mountain landscapes remain in a fragile balance between excess and shortage of water resources as well as underlying salinity levels and other soil properties (Song et al. 2022; Sagwal et al. 2023). Although the future of the alpine halophiles is at a crossroads, the direction of its prospective population changes would depend heavily on complex hydrological and edaphic changes across the mountain ecosystem.

## supplementary material

Data available from the Dryad Digital Repository: https://doi.org/10.5061/dryad.sn02v6x85 [https://datadryad.org/stash/share/6Qvb_9E148Mtonvnf-2ygEI0g--NCwzxGMPiRtlUBtg].

## Data Availability

Voucher specimens of *Puccinellia pamirica* can be accessed through the herbarium of the Jagiellonian University, Kraków, Poland (KRA). Additional materials associated with this article are available from the Dryad Digital Repository: Supplementary Appendix S1—Research Area, Model System and Sampling; Supplementary Appendix S2—SNP Marker Discovery and Genotyping via the DArTseq Platform; Supplementary Appendix S3—PCoA, Population Structure, Fixed and Private Alleles; Supplementary Appendix S4—Treemix; Supplementary Appendix S5—Stairway Plot, DIYABC-RF; Supplementary Appendix S6—MaxEnt Ecological Niche Modeling; Supplementary Appendix S7—Isolation Factors and Linear Mixed-Effects Models. Raw SNP genetic dataset in the dartR (genlight) format, MaxEnt input layers, MaxEnt output raster files, as well an input dataset for Linear Mixed-Effects Models are also available from the Dryad Digital Repository.
